# Cancer Patients’ Experiences and Management of Chemotherapy-Induced Peripheral Neuropathy: A Qualitative Study in Qassim, Saudi Arabia

**DOI:** 10.3390/healthcare14070902

**Published:** 2026-03-31

**Authors:** Dhay Abdullah Alharbi, Riad Abdulrahman Albeheeji, Layan Saleh Alzeghaibi, Aryam Abdullah Alabody, Norah Abdullah Aljutayli, Maryam Farooqui, Bader Alshamsan, Abdulkarim Alharbi, Ahmad Alfawazi, Norah Alodhaybi, Saud Alsahali

**Affiliations:** 1 Department of Pharmacy Practice, College of Pharmacy, Qassim University, Buraidah 51452, Qassim, Saudi Arabia; daiabduallah@gmail.com (D.A.A.); riadalbeheeji@gmail.com (R.A.A.); layansaleh081@gmail.com (L.S.A.); aryam.23jj@gmail.com (A.A.A.); norahaljutaily@gmail.com (N.A.A.); s.alsahali@qu.edu.sa (S.A.); 2 Discipline of Social and Administrative Pharmacy (DSAP), School of Pharmaceutical Sciences, Universiti Sains Malaysia, Gelugor 11800, Malaysia; 3 Department of Medicine, College of Medicine, Qassim University, Buraydah 52571, Saudi Arabia; bshmsan@qu.edu.sa; 4 Oncology Pharmacy Department, Prince Faisal Cancer Center, Qassim Health Cluster, Buraidah 52367, Saudi Arabia; ph0abdulkarim@gmail.com; 5 Department of Hematology, Prince Faisal Cancer Center, Qassim Health Cluster, Buraidah 52367, Saudi Arabia; aa.alfawazi@gmail.com; 6 Oncology Nursing Department, Prince Faisal Cancer Center, Qassim Health Cluster, Buraidah 52367, Saudi Arabia; nouraodhaibi@gmail.com

**Keywords:** cancer, CIPN, qualitative study, awareness, symptoms, chemotherapy

## Abstract

**Background/Objectives**: Chemotherapy-induced peripheral neuropathy (CIPN) is a painful, debilitating condition that significantly impairs patient quality of life and often necessitates dose modification or discontinuation of chemotherapy, which can adversely impact patient outcomes and overall survival. This study aims to explore the experiences of cancer patients affected by CIPN and identify the challenges encountered in managing this condition. **Methods**: Data were collected through qualitative semi-structured interviews with 20 cancer patients with confirmed CIPN. The semi-structured interviews were held between April and June 2025 at a cancer center in the Qassim Region, Saudi Arabia, and were audio-recorded, transcribed, and analyzed using thematic analysis following Braun and Clarke. **Results**: Patients reported experiencing a considerable burden of CIPN symptoms, particularly during the early phases of chemotherapy, with some reporting gradual changes over time. Symptom unpredictability was reported across different types of cancer and regimens, regardless of age or gender. Sensory disruptions and functional impairments were prominent among many participants. Patients with higher levels of education, including those with family members in healthcare, demonstrated a stronger understanding of their condition and treatment explanations. Across cancer groups, patients expressed dissatisfaction with the prescribed therapies’ side effects. A subset of patients expressed a strong willingness to participate in clinical trials. **Conclusions**: The findings highlight the need for improved patient education, early symptom recognition, and comprehensive supportive care strategies. Healthcare providers should proactively address CIPN in treatment discussions and offer tailored interventions that go beyond physical symptoms. Additionally, further research is needed to identify and prevent CIPN across diverse populations.

## 1. Introduction

Cancer remains a major contributor to global morbidity and mortality. In 2020, it accounted for approximately 10 million deaths worldwide, representing nearly one in six deaths globally. Despite significant advances in oncology, cytotoxic chemotherapy remains a cornerstone of treatment for many malignancies at all disease stages [1,2,3,4]. Chemotherapy is widely used in cancer treatment, which effectively targets rapidly proliferating cancer cells. However, it inadvertently impacts healthy cells [5,6,7]. This unavoidable consequence results in a variety of conditions that can significantly influence patients’ physical health, quality of life (QOL), and overall well-being [7]. Consequently, chemotherapy-related toxicities remain a major concern for both patients and clinicians despite advancements in survival rates [8]. The nervous system is particularly vulnerable to chemotherapeutic toxicity, which may result in several forms of neuropathy [2]. Among these, chemotherapy-induced peripheral neuropathy (CIPN) is one of the most common and clinically significant complications [2].

CIPN is a painful, debilitating condition that significantly impairs QOL and often necessitates dose modification or discontinuation of chemotherapy, which can adversely impact patient outcomes and overall survival [9]. It is characterized by a group of neuromuscular symptoms stemming from nerve damage and predominantly affecting the hands and feet, including numbness, tingling, pain, muscle weakness, cold sensations, and motor or autonomic nerve manifestations, such as balance disturbances and dizziness [10]. The incidence of CIPN varies significantly, and it is influenced by the specific chemotherapeutic agent used, the administered dose, and the duration of exposure [9]. Notably, individuals receiving platinum-based agents (cisplatin, carboplatin, and oxaliplatin) and taxanes (paclitaxel and docetaxel) exhibit the highest rates of chronic moderate-to-severe CIPN or painful CIPN, with prevalence estimates of 44.47% and 55.68%, respectively [9]. Approximately 70% of patients experience CIPN within the initial month of treatment, 60.0% at three months, and 30.0% at six months [9]. Multiple studies indicate that while many patients attain remission from cancer, they may exhibit persistent symptoms of CIPN, resulting in serious challenges that considerably limit their daily life activities, including walking, engaging in hobbies, and maintaining relationships [11,12]. This highlights a significant need for pain management interventions to address these side effects of cancer treatment.

Managing CIPN presents a considerable challenge for healthcare providers, as underscored by the American Society of Clinical Oncology (ASCO) and the European Society for Medical Oncology (ESMO) [2,9,13]. Current therapeutic strategies predominantly encompass pharmacological interventions, such as duloxetine, pregabalin, amitriptyline, and gabapentin [13,14,15,16,17], as well as non-pharmacological approaches, including acupuncture, massage, exercise, and dietary supplements [14,18,19,20]. Furthermore, some patients have reported taking vitamin B, calcium, and magnesium infusions as adjunctive measures to relieve symptoms [14]. However, limited clinical evidence supports the efficacy of these interventions [9,13], and the FDA has not approved any agents specifically for the prevention of CIPN [20]. This prevailing lack of effective treatment options underscores a critical gap in CIPN management and the pressing need for further research to advance therapeutic strategies in this area.

Within the Saudi context, cancer patients’ understanding of CIPN symptoms, cultural beliefs, health care seeking behaviors, family support, and the use of complementary medicines may influence how patients perceive and manage such symptoms. However, in-depth evidence is limited, especially among cancer patients from the Qassim region. This gap limits the development of culturally appropriate cancer care strategies. The present research explored the experiences of cancer patients affected by CIPN in Al-Qassim Region, Saudi Arabia. By identifying the challenges encountered in managing this condition, the study aims to enhance patient education and treatment outcomes. A comprehensive understanding of these experiences is essential for developing targeted interventions that effectively address the needs of both patients and healthcare providers.

## 2. Methodology

### 2.1. Study Design

Qualitative semi-structured interviews were conducted to obtain a comprehensive understanding of patient perspectives regarding CIPN. The qualitative design was chosen to enable the exploration of insights and provide an in-depth understanding, which quantitative designs may not adequately capture. The manuscript was prepared in accordance with the Consolidated Criteria for Reporting Qualitative Research (COREQ) checklist [21]. Participants were informed that their participation was entirely voluntary, their information would remain strictly confidential, and that they could withdraw from the interview at any point without consequences. All participants provided written informed consent. Qassim Health Cluster granted ethical approval to the study (607-46-7335 on 25 December 2024).

### 2.2. Participants and Setting

Participants were recruited from Prince Faisal Cancer Centre in Qassim Region, Saudi Arabia. Eligibility criteria included age 18–90 years; confirmed diagnosis of CIPN by the medical oncologists using Common Terminology Criteria for Adverse Events (CTCAE) Version 5.0; receipt of chemotherapy within the past year; and willingness to participate with provision of informed consent. Patients whose last chemotherapy cycle was more than one year prior to interview were excluded to minimize recall bias.

A purposive sampling strategy was used to recruit participants with diverse cancer types and chemotherapy experiences. Eligible patients were identified through clinic lists and approached by the researcher during routine oncology visits.

### 2.3. Data Collection and Saturation

Semi-structured interviews were conducted between April 2025 and June 2025, with each interview lasting approximately 15–30 min. The semi-structured format captured detailed insights into how patients cope with CIPN, their perspectives on its management, and the challenges they face. The interview guide was developed after an extensive literature search with open-ended questions to explore participants’ experiences in greater depth [2,10]. The initial draft was discussed among the experts from the Faculty of Pharmacy, Qassim University, Saudi Arabia. Furthermore, discussions with public health experts and oncologists to refine the interview guide were conducted. Pretesting of the interview guide was performed but data from the pilot study were not added to the final analysis. Interviews were conducted in Arabic, the participants’ native language, and subsequently transcribed and translated into English by a bilingual researcher. Back-translation was then performed by an independent translator to ensure accuracy. The translated text was cross-checked against the original Arabic scripts to preserve meaning. Interviews were conducted by a trained researcher in a private room at the cancer center to ensure comfort and confidentiality. All interviews were audio-recorded to ensure accuracy and prevent the loss of important information during transcription. Patients’ demographic and disease-related data were obtained by a questionnaire attached to the patient information sheet. All interviews were audiotaped so that verbatim transcriptions could be created. Each interview was transcribed verbatim. The transcripts were sent to the participants for approval. Data collection continued until data saturation was achieved, defined as the point at which no new themes or subthemes had emerged in the previous three interviews. Thematic redundancy was monitored across participants with diverse cancer types and chemotherapy regimens, and no substantially new themes or subthemes emerged in the final interviews. Accordingly, the sample size was considered sufficient to capture the range and depth of patient experiences while maintaining the rigor of qualitative analysis.

### 2.4. Data Analysis

Data were analyzed using inductive reflexive thematic analysis (RTA) as outlined by Braun and Clarke [22]. RTA was chosen because it facilitated the generation of rich insights into participants’ perspectives, lived experiences, and the potential barriers encountered in managing CIPN. Transcripts were read repeatedly to achieve familiarization, followed by systematic coding of meaningful segments. Coding was conducted iteratively across the dataset, with codes refined through ongoing comparison and discussion. Codes were then collated into candidate themes, which were reviewed and refined to ensure coherence, internal consistency, and a clear distinction between themes. Final themes were defined and named to reflect participants’ experiences and perspectives. The analysis was conducted manually following repeated, in-depth readings of all interview transcripts. The research team consisted of investigators with backgrounds in pharmacy and healthcare. The researchers had no prior clinical relationship with the participants. Reflexivity was maintained throughout the analytic process through regular discussions among the research team to reflect on coding decisions, theme development, and the potential influence of researchers’ perspectives on data interpretation.

## 3. Results

A total of 20 patients participated in the study. Most participants were female (*n* = 15) and aged 40 years or older. The median age was 52.5 years (range 25–69 years). Breast cancer was the most common diagnosis (8/20), followed by colorectal cancer (6/20), and endometrial cancer (2/20). Lung, pancreatic, peritoneal carcinoma, and testicular cancers were each represented by one participant. Table 1 summarizes participants’ chemotherapy regimens, treatment duration (3–12 months), and number of treatment cycles (ranging from 3 to over 34).

The qualitative analysis identified two key categories: experiences of CIPN and perspectives on its management. Each category comprised several themes and subthemes as summarized in Table 2. Representative quotations are presented using anonymized patient identifiers (P1–P20).

### 3.1. Category 1: Experiences with CIPN

#### 3.1.1. Physical and Sensory Experiences

##### Unpredictable Sensory Symptoms

Symptom unpredictability was reported across different types of cancer and regimens, regardless of age or gender. Both breast and colorectal cancer patients frequently described sensory disruptions such as numbness, tingling, burning sensations, and cold sensitivity. In some cases, symptoms worsened with cumulative chemotherapy cycles, whereas in others they appeared abruptly after only a few sessions. This variability generated uncertainty among patients, complicating their ability to cope.

##### Loss of Function

Functional impairments were prominent among many participants. Patients described difficulties in walking, climbing stairs, maintaining balance, and performing fine motor tasks, such as holding objects or opening bottles. For younger participants, these limitations were especially distressing due to the sudden disruption of previously active lifestyles. Several older patients transitioned to wheelchair use due to severe mobility loss, illustrating the debilitating effects of CIPN across treatment regimens.

##### Pain and Discomfort

Pain manifestations extended beyond neuropathic numbness to include burning, cramping, and musculoskeletal fatigue. Pancreatic and colorectal cancer patients undergoing intensive regimens, such as FOLFIRINOX or FOLFOX, reported particularly severe pain, whereas some breast cancer patients described skin hypersensitivity and inflammation. Despite pharmacological and supportive interventions, relief was often partial and inconsistent.

##### Tolerance

Tolerance to neuropathic symptoms varied. Some patients, particularly those in advanced stages (e.g., stage IV colorectal or breast cancer), expressed their willingness to endure symptoms to continue treatment, emphasizing the priority of survival. Conversely, others regretted continuing chemotherapy due to overwhelming symptoms that significantly compromised daily functioning. This divergence reflects the complex trade-offs patients face between QOL and managing cancer progression.

#### 3.1.2. Emotional and Psychological Impact

##### Anxiety and Depression

Anxiety and depression were recurrent themes reported by many participants, irrespective of cancer type or treatment regimen. Emotional distress ranged from mild frustration to clinically concerning depressive episodes. Patients described feelings of helplessness linked to physical restrictions as well as social withdrawal due to embarrassment caused by tremors, imbalance, or visible neuropathic symptoms. Some employed faith-based resilience to cope, whereas others viewed treatment as emotionally exhausting. A lack of communication with oncologists about side effects further aggravated psychological distress.

#### 3.1.3. Health Literacy and Support Network

##### Awareness Level

Patients with higher levels of education, including those with family members in healthcare, demonstrated a stronger understanding of their condition and treatment explanations, which facilitated a better ability to follow medical recommendations and cope with neuropathic side effects. By contrast, less-informed participants and those with minimal medical exposure struggled to interpret dietary advice, medication management instructions, and symptom terminology, reinforcing disparities in health literacy.

##### Role of Family Members and Healthcare Providers

Family members, particularly those with healthcare backgrounds, played a vital role in offering reassurance, guidance, and symptom management strategies. However, a recurring concern across patients was insufficient information from healthcare providers, with many participants reporting that doctors discussed nausea and fatigue but not neuropathy prior to starting treatment. Participants associated this limited preparatory information with feelings of unpreparedness, frustration, and—in some cases—loss of trust in oncology care.

### 3.2. Category 2: Perceptions of CIPN Management

#### 3.2.1. Perceived Effectiveness of CIPN Management

##### Effectiveness

Perceptions of treatment effectiveness varied greatly. While some patients reported modest improvement from vitamins, chemotherapy schedule adjustments, or medications such as nerve stabilizers, many emphasized that neuropathic symptoms persisted. Supportive measures such as physical rest, dietary modifications, and family care were recognized as partially useful, but none were perceived as truly curative.

##### Side Effects

Across cancer groups, patients expressed dissatisfaction with prescribed therapies’ side effects. Commonly reported adverse effects included headaches, gastrointestinal complaints, and severe fatigue. For several individuals, particularly those on aggressive regimens, side effects added an additional layer of suffering beyond neuropathy itself. This dual burden compromised trust in pharmacological management strategies.

#### 3.2.2. Willingness to Participate in Clinical Trials for New Treatments

##### Openness to Participation

A subset of patients expressed strong willingness to engage in clinical trial opportunities, with motivations ranging from personal hope for symptom relief to an altruistic desire to advance treatment for future patients. Younger participants and those with less effective treatment histories were more open to participating in experimental protocols.

##### Concern and Reservations

In contrast, some patients from both the breast and colorectal groups expressed hesitancy about trials, citing fatigue from prolonged treatment regimens and fears of additional side effects. For a few, the sentiment of “already suffering enough” dampened enthusiasm for further experimentation. This ambivalence highlights the importance of addressing patients’ emotional readiness and providing detailed counseling on risks and benefits when integrating them into new research protocols.

## 4. Discussion

This study explored cancer patients’ lived experiences of CIPN and identified potential barriers to CIPN management among cancer patients in the Qassim Region [2]. Patients described CIPN as a substantial and multifaceted burden, particularly during the early phases of chemotherapy, although some reported gradual symptom evolution over time. These findings align with prior research indicating considerable variability in CIPN onset, severity and progression [23]. Consistent with existing evidence, platinum-based agents and taxanes were frequently associated with neuropathic symptoms among our participants [24]. Platinum compounds primarily affect sensory neurons in the dorsal root ganglia, whereas taxanes are associated with cumulative peripheral nerve toxicity [25]. This provides important context for our findings, as participants receiving oxaliplatin- or taxane-containing regimens commonly described numbness, pain, burning sensations, and functional limitations. Patients receiving combination or more intensive regimens also appeared to report a greater overall symptom burden, suggesting that treatment intensity and cumulative exposure may influence how CIPN is experienced in daily life.

CIPN is widely recognized as a dose- and agent-dependent toxicity related to the neurotoxic effects of several chemotherapeutic drugs [3]. The persistence and fluctuating intensity of symptoms described by participants mirror reports that CIPN can endure long after treatment completion and substantially impair quality of life [2,26].

Multiple factors influencing the severity and duration of patients’ peripheral neuropathy have been described in the literature, including the chemotherapeutic agent, the dosage administered, and the cancer stage at diagnosis. While neuropathy can persist long term, its intensity may diminish over time [26]. Several participants in our study described persistent or fluctuating symptoms, consistent with prior reports. Studies show that CIPN can lead to functional limitations, with about 13% of patients experiencing difficulties in daily activities and nearly 21% becoming reliant on others due to neurotoxicity. Overall, CIPN adversely affects QOL in approximately 49% of patients [2].

CIPN symptoms were described by participants as difficult to manage, causing physical and emotional distress, impairing functional abilities and social roles, and significantly limiting patients’ daily lives [27]. The major symptoms of CIPN include numbness, tingling, burning sensations, pain in the upper and lower extremities, and altered sensitivity, such as hypoesthesia or hyperesthesia. In addition, patients often experience difficulties in motor function, such as muscle weakness and spasms, which can disrupt both fine and gross motor skills, increase the risk of injury, and further compromise their overall QOL [28]. These symptoms were perceived to limit engagement in routine tasks, such as walking, dressing, and writing. The complex impact of CIPN often persists long after treatment ends, making it difficult for patients to adapt to their altered daily lives [2]. Health literacy together with cultural factors may further influence how cancer patients perceive these symptoms and seek help. The perceived severity of chemotherapy-induced side effects may also affect patients’ decision to continue or forgo cancer treatments [29]. In the Saudi context, strong family support, which is characteristic of the local culture, may play an important role in helping patients cope with treatment-related side effects, maintain daily activities, and preserve social engagement [30].

Our interviews with cancer patients revealed that, for most, little emphasis had been placed on discussing or explaining CIPN symptoms. As in previous studies, patients reported limited awareness of CIPN, which often received lower priority than other chemotherapy-related adverse effects [31]. Experiencing CIPN shaped patients’ perceptions and enhanced their awareness of the condition, emphasizing the need to provide comprehensive CIPN information before starting chemotherapy and to reinforce it throughout and after treatment [2]. Several patients expressed regret about undergoing chemotherapy, suggesting that awareness of potential side effects may have influenced their decision to decline treatment. This highlights the challenge of balancing comprehensive patient education with the risk of refusing treatment. Consistent with our findings, the perceived severity of side effects may influence treatment decision-making and shape patients’ willingness to continue chemotherapy. In this context, oncology healthcare professionals may play a pivotal role in early screening for treatment-related side effects and in providing patient education on coping strategies and supportive symptom management. Routine CIPN screening during chemotherapy may also help improve early symptom recognition, encourage timely reporting, and support more responsive clinician–patient communication.

Despite some regret, many patients accepted the side effects and actively sought strategies to manage them, reflecting resilience and adaptive coping. Participants in this study also sought non-pharmacological remedies to cope with CIPN, such as acupuncture, yoga, and maintaining a positive mindset. The participants reported using tramadol as a pharmacological approach to manage CIPN-related pain. Although it offered some relief, several patients experienced side effects, notably headaches, which added to their discomfort and adversely affected their QOL. Complementary and alternative medicine (CAM) therapies, known for being minimally invasive and typically associated with low risk of toxicity, have been integrated into clinical practice as supportive approaches for managing pain. Systematic reviews indicate that these therapies can enhance various aspects of well-being in cancer patients and survivors, including improved QOL, better sleep, enhanced mood, and reduced stress and anxiety. Nonetheless, there remains a lack of strong evidence confirming their specific effectiveness in managing cancer-related pain [32].

The study’s participants varied in their willingness to participate in clinical trials for new CIPN treatments, with some ready to participate and others hesitant. This finding highlights the need to consider patient attitudes and preferences when designing future clinical trials, as patients must first be aware that clinical trials are an available treatment option and understand the nature of clinical research before making an informed decision. Notably, a study in Saudi Arabia found that only 51.2% of cancer patients were aware of clinical trials as a treatment option, and concerns about side effects and lack of information were among the main barriers to participation [33]. These findings emphasize that patients must first be informed that clinical trials are available and must understand the nature of clinical research before they can make truly informed decisions.

Although numerous studies have investigated potential treatments for CIPN, most agents bring their own toxicity concerns, leading to their removal from ASCO and ESMO guidelines. For patients with established painful neuropathy, duloxetine may be considered after a thorough evaluation of the CIPN presentation and individual risk factors. Evidence indicates that duloxetine can reduce CIPN-related pain, and exploratory findings suggest that it may alleviate non-painful symptoms [2]. Furthermore, secondary and exploratory analyses of the duloxetine trial indicated that patients with better baseline emotional health (less worry, tension, irritability, and depression) were approximately four times more likely to respond to treatment. This finding suggests that individuals whose pain occurs as part of a broader symptom cluster may experience less benefit from duloxetine [34]. Recent evidence has evaluated the role of gabapentinoids in the management of CIPN. A 2023 systematic review and meta-analysis examined multiple studies assessing pregabalin and gabapentin for both prevention and treatment of CIPN; the analysis found that these agents demonstrated limited efficacy in preventing CIPN and only modest improvements in pain reduction, with some benefit observed in QOL measures [16]. Overall, both drugs were generally well tolerated, with mild adverse effects, such as dizziness and somnolence. These findings suggest that, although pregabalin is sometimes used clinically for CIPN, the current evidence does not support its routine use for robust pain control or prevention, highlighting the need for further high-quality trials.

To date, multiple preclinical studies have investigated treatments for neuropathic pain. Notably, recent research has focused on the role in CIPN of 5-HT3 receptors and their antagonists. In animal models, intrathecal administration of the 5-HT3 receptor antagonist ondansetron significantly reduced mechanical and cold hypersensitivity in paclitaxel-treated animals. Interestingly, lower doses of ondansetron showed antinociceptive effects in the von Frey test but not in the cold plate test. These findings suggest that 5-HT3 receptor blockade may represent a promising strategy for managing chemotherapy-induced neuropathic pain. Similarly, another study examined the therapeutic potential of tropisetron, demonstrating that intrathecal administration in rats with spinal cord clip compression injury reduced hyperalgesia and mechanical allodynia, although cold allodynia remained unaffected. This indicates a possible clinical application of 5-HT3 antagonists for treating central neuropathic pain, particularly in patients experiencing hyperalgesia and tactile allodynia [35].

## 5. Limitations

This study had a predominance of female participants, most of whom were over 40 years old and diagnosed with breast cancer. This demographic imbalance may limit the transferability of the findings to male patients, younger individuals, and those with other cancer types. Additionally, as a qualitative study conducted in a single region, the findings may reflect context-specific experiences that are not fully transferable to other settings. The relatively small sample size, although appropriate for qualitative research, may not capture the full range of CIPN experiences. Furthermore, although participants were recruited within one year of chemotherapy to reduce recall bias, some degree of recall inaccuracy may still be present when reporting symptom onset and progression. Although CIPN was clinically confirmed, severity was not systematically incorporated into study analysis. Nevertheless, the qualitative data provided a rich description of the physical, social, and emotional impact of these side effects on patients’ well-being.

## 6. Conclusions

This study provides important insights into how deeply CIPN affects cancer patients. Many patients struggle with pain, weakness, and emotional distress, often without adequate guidance or support. Those with better health knowledge and strong support systems appeared to cope more easily. While patients were open to new treatment options, including experimental ones, gaps in communication and access to care remain major hurdles. These findings highlight the need for improved patient education, early symptom recognition, and comprehensive supportive care strategies. Healthcare providers should proactively address CIPN in treatment discussions and offer tailored interventions that go beyond physical symptoms. Additionally, further research is needed to identify more effective strategies to prevent and treat CIPN in diverse patient populations.

## Figures and Tables

**Table 1:** Demographic and clinical characteristics of study participants.

Patient Number	Age	Gender	Type of Cancer	Stage of Cancer	Duration of Chemo	Protocol Name	Number of Cycles
P1	50	Female	Endometrial cancer	IV	>12 months	Doxorubicin + dacarbazine → Docetaxel + gemcitabine	3 cycles → 8 cycles
P2	41	Female	Lung cancer	IV	3–6 months	Gemcitabine + carboplatin → Docetaxel	3 cycles → 2 cycles
P3	53	Female	Endometrial cancer	IV	3–6 months	Paclitaxel + carboplatin	4 cycles
P4	25	Male	Testicular cancer	II	3–6 months	BEP (bleomycin/etoposide/cisplatin)	3 cycles
P5	52	Female	Breast cancer	III	3–6 months	Paclitaxel + carboplatin + pembrolizumab → AC + pembrolizumab	4 cycles → 4 cycles
P6	52	Female	Breast cancer	IV	6–12 months	AC (doxorubicin/cyclophosphamide) → Paclitaxel + HP (trastuzumab + pertuzumab)	4 cycles AC → 12 weekly paclitaxel with HP
P7	46	Female	Breast cancer	II	6–12 months	AC → Docetaxel	4 cycles → 3 cycles
P8	56	Male	Pancreatic cancer	IV	>12 months	FOLFIRINOX → FOLFIRI	6 cycles → 34 cycles
P9	66	Male	Colorectal cancer	III	6–12 months	XELOX → FOLFOX	2 cycles → 7 cycles
P10	59	Female	Colorectal cancer	III	>12 months	XELOX	8 cycles
P11	60	Female	Colorectal cancer	III	6–12 months	FOLFOX	11 cycles
P12	52	Female	Breast cancer	IV	>12 months	AC → Weekly paclitaxel	4 cycles → 8 weekly paclitaxel
P13	69	Female	Peritoneal carcinomatosis	III	6–12 months	Paclitaxel + carboplatin → Liposomal doxorubicin	6 cycles → 2 cycles
P14	54	Female	Colorectal cancer	III	6–12 months	XELOX	4 cycles
P15	54	Female	Breast cancer	II	3–6 months	TCHP (docetaxel/carboplatin/trastuzumab/pertuzumab) → HP	6 cycles → 9 cycles
P16	60	Female	Breast cancer	III	3–6 months	AC → THP (docetaxel/trastuzumab/pertuzumab)	4 cycles → 3 cycles
P17	46	Female	Breast cancer	II	3–6 months	TCHP → HP	6 cycles → 8 cycles
P18	46	Female	Breast cancer	II	3–6 months	AC → THP	4 cycles → 3 cycles
P19	58	Male	Colorectal cancer	IV	>12 months	XELOX + bevacizumab → XELIRI	14 cycles → 21 cycles
P20	39	Male	Colorectal cancer	IV	>12 months	FOLFOX → FOLFIRI	12 cycles → 3 cycles

Abbreviations: AC (doxorubicin + cyclophosphamide); BEP (bleomycin + etoposide + cisplatin); FOLFIRI (5-FU + leucovorin + irinotecan); FOLFOX (5-FU + leucovorin + oxaliplatin); XELOX (capecitabine + oxaliplatin); XELIRI (capecitabine + irinotecan); TCHP (docetaxel + carboplatin + trastuzumab + pertuzumab); THP (docetaxel + trastuzumab + pertuzumab); HP (trastuzumab + pertuzumab).

**Table 2:** Categories, themes, subthemes, and representative quotations from thematic analysis.

Category	Theme	Subtheme	Quotation
Experiences with CIPN	Physical and sensory experiences	Unpredictable sensory symptoms	“It’s one of the hardest things. Losing sensation due to numbness is very difficult.” (P20) “Numbness comes and goes and tingling too. But the tremor is 24/7.” (P4) “If I hold something, my hand locks in that position.” (P19)
		Loss of function	“I can’t walk without assistance … my balance is lost.” (P16) “I can’t even go to the bathroom by myself.” (P12). “Yes, my leg movement is difficult. I can’t walk much. My nerves and strength are gone.” (P20)
		Pain and discomfort	“When I walk, it feels like the skin is peeled off, and it hurts.” (P20) “Sometimes my nerves hurt—like when I want to do something, my nerves can’t handle it.” (P16) “The internal pain is worse.” (P6) “In my fingertips and feet, I feel a burning sensation.” (P9)
		Tolerance	“The symptoms can be reduced by lowering the dose, but I prefer not to reduce it. I ask them to keep it the same because I can tolerate it.” (P1) “I can tolerate the pain.” (P5) “They said it might be severe, but when I experienced it, it was concerning yet tolerable.” (P17) “At first I was scared, but now I’m used to it.” (P16) “God willing, it’s just a phase and will pass.” (P14)
	Emotional and psychological impact	Depression and anxiety	“More like sadness or emotional upset, but I try to ignore it.” (P9) “Every time I experienced something new, it affected me psychologically. The doctor would say, ‘It is normal’” (P7) “I went through a period of depression. I stopped going out and only went between the hospital and home.” (P11)
	Health literacy and support network	Awareness level	“When I research, I find studies in English, and my English is only at a high school level.” (P11) “I may have read, but I didn’t focus on the numbness and related symptoms.” (P18) “I looked up other women’s experiences on TikTok, and that’s how I learned about it.” (P17)
		Role of family members and healthcare providers	“Two of my brothers are pharmacists, and many family members work in healthcare, so I understand well.” (P1) “He mentioned numbness, but I didn’t expect it to happen.” (P10) “He didn’t explain how chemo would affect my overall health. If I had known, I might not have agreed.” (P6) “Most of what I know is from the internet. My father’s friend, an oncology consultant, gave me some information.” (P4)
Perspectives on CIPN management	Perceived effectiveness of CIPN management	Effectiveness	“Only slightly, but I continue to take them.” (P7) “With the medication, it’s better.” (P3) “The symptoms are still there but have improved a lot.” (P4) “Yes, especially the painkillers.” (P6)
		Side effects	“It caused me a continuous headache. They said it works on the brain. I also felt anxiety, but it reduced the pain.” (P2) “Terrible. I got every side effect.” (P6)
	Willingness to participate in clinical trial for new treatment	Agreeing to participate	“I’m willing to be a test subject if it helps. I just want someone to hear my voice. I have no problem with that.” (P8) “Sure, in my current condition, I’d like to try anything.” (P11) “I’ve finished treatment now, but if I were still doing chemo, absolutely. I’d join to help spare others what I went through.” (P6)
		Concern and reservations	“What I am already suffering is enough.” (P1) “I wouldn’t take something untested.” (P12) “I’m now afraid of side effects.” (P10)

## Data Availability

The raw data supporting the conclusions of this article will be made available by the authors on request.
